# Loss of basal feet on Odf2 deletion perturbs polarization of basal bodies

**DOI:** 10.1186/2046-2530-1-S1-O26

**Published:** 2012-11-16

**Authors:** K Kunimoto, YY Yamazaki, TN Nishida, KS Shinohara, HI Ishikawa, TH Hasegawa, TO Okanoue, HH Hamada, TN Noda, AT Tamura, ST Shoichiro Tsukita

**Affiliations:** 1 Laboratory of Biological Science, Graduate School of Frontier Biosciences and Graduate School of Medicine, Osaka University, Japan; 2 Research Center for Ultra-high Voltage Electron Microscopy, Osaka University, Japan; 3 Developmental Genetics Group, Graduate School of Frontier Biosciences, Osaka University and CREST, Japan Science and Technology Corporation (JST), Japan; 4 Department of Biochemistry and Biophysics, University of California, San Francisco, USA; 5 Department of Hepatology, Saiseikai Suita Hospital, Osaka, Japan; 6 Department of Cell Biology, Cancer Institute of Japanese Foundation for Cancer Research, Tokyo, Japan; 7 Department of Cell Biology, Faculty of Medicine, Kyoto University, Japan

## 

Synergic multiciliary beating relies on cilia generated from basal bodies, with which basal feet are regularly associated through molecular mechanisms that remain unknown. Here we show that the coordinated multiciliary action is disturbed in *Odf2* mutant mice, resulting in primary ciliary dyskinesia and a characteristic coughing/sneezing-like phenotype. *Odf2*-deficiency depleted basal feet from basal bodies to perturb the planar cell polarity (PCP) of basal bodies, as shown by ultra-high voltage electron microscopic tomography (UHVEMT) of wild and *Odf2* mutant tracheas. The apical microtubular lattice, which is organized by the keystone positioning of basal feet/basal bodies, was lost in *Odf2*-mutant animals, irrespective of normal localization of Vangl1, the PCP core protein. These findings demonstrate that Odf2 is required for the formation of basal feet. Odf2-based basal feet play a critical role in the PCP-based arrangement of the microtubular lattice and basal bodies, thereby enabling coordinated multiciliary beating.

